# Radiomics models based on multisequence MRI for predicting PD-1/PD-L1 expression in hepatocellular carcinoma

**DOI:** 10.1038/s41598-023-34763-y

**Published:** 2023-05-12

**Authors:** Xue-Qin Gong, Ning Liu, Yun-Yun Tao, Li Li, Zu-Mao Li, Lin Yang, Xiao-Ming Zhang

**Affiliations:** 1 Medical Imaging Key Laboratory of Sichuan Province, Interventional Medical Center, Department of Radiology, Medical Research Center, Affiliated Hospital of North Sichuan Medical College, Nanchong, 637000 China; 2 Department of Radiology, Chongqing University Cancer Hospital, Chongqing, 400030 China; 3 Department of Pathology, Affiliated Hospital of North Sichuan Medical College, Nanchong, 637000 China

**Keywords:** Biomarkers, Gastroenterology

## Abstract

The purpose of this study was to explore the effectiveness of radiomics based on multisequence MRI in predicting the expression of PD-1/PD-L1 in hepatocellular carcinoma (HCC). One hundred and eight patients with HCC who underwent contrast-enhanced MRI 2 weeks before surgical resection were enrolled in this retrospective study. Corresponding paraffin sections were collected for immunohistochemistry to detect the expression of PD-1 and PD-L1. All patients were randomly divided into a training cohort and a validation cohort at a ratio of 7:3. Univariate and multivariate analyses were used to select potential clinical characteristics related to PD-1 and PD-L1 expression. Radiomics features were extracted from the axial fat-suppression T2-weighted imaging (FS-T2WI) images and the arterial phase and portal venous phase images from the axial dynamic contrast-enhanced MRI, and the corresponding feature sets were generated. The least absolute shrinkage and selection operator (LASSO) was used to select the optimal radiomics features for analysis. Logistic regression analysis was performed to construct single-sequence and multisequence radiomics and radiomic-clinical models. The predictive performance was judged by the area under the receiver operating characteristic curve (AUC) in the training and validation cohorts. In the whole cohort, PD-1 expression was positive in 43 patients, and PD-L1 expression was positive in 34 patients. The presence of satellite nodules served as an independent predictor of PD-L1 expression. The AUC values of the FS-T2WI, arterial phase, portal venous phase and multisequence models in predicting the expression of PD-1 were 0.696, 0.843, 0.863, and 0.946 in the training group and 0.669, 0.792, 0.800 and 0.815 in the validation group, respectively. The AUC values of the FS-T2WI, arterial phase, portal venous phase, multisequence and radiomic-clinical models in predicting PD-L1 expression were 0.731, 0.800, 0.800, 0.831 and 0.898 in the training group and 0.621, 0.743, 0.771, 0.810 and 0.779 in the validation group, respectively. The combined models showed better predictive performance. The results of this study suggest that a radiomics model based on multisequence MRI has the potential to predict the preoperative expression of PD-1 and PD-L1 in HCC, which could become an imaging biomarker for immune checkpoint inhibitor (ICI)-based treatment.

## Introduction

Hepatocellular carcinoma (HCC) is one of the most common malignant tumors and imparts a heavy disease burden^1,2^. To date, the prognosis of HCC has remained poor^3,4^. Therefore, new treatment methods need to be explored. In recent years, immune checkpoint inhibitor therapy for HCC has attracted much attention^5–9^. Drugs blocking the programmed cell death 1 (PD-1) and programmed death ligand 1 (PD-L1) pathways have shown excellent effectiveness and safety, bringing new hope to HCC patients^7^. PD-1 is a member of the CD28 immunoglobulin superfamily, is expressed in T lymphocytes, and has a negative regulatory effect on antigen responses^10,11^. PD-L1, the main ligand of PD-1, is broadly expressed in antigen-presenting cells and tumor cells^11^ and induces T-cell apoptosis or dysfunction after it binds to PD-1, eventually leading to tumor immune escape^12^. Previous studies have shown that the expression status of PD-1/PD-L1 in tumours is associated with treatment responses and clinical outcomes following PD-1/PD-L1 pathway inhibition^13–21^ and can be used as a biomarker for predicting the effectiveness of ICI treatment^22,23^. The main challenge lies in selecting the patient subgroup that would benefit most and avoid ineffective treatment resulting from blocking the PD-1/PD-L1 pathway. Therefore, it is crucial to evaluate the expression status of PD-1/PD-L1 in HCC patients before treatment. However, PD-1/PD-L1 detection currently mainly depends on the immunohistochemical methods involving pathological tissue acquired from resection or biopsies. There is an urgent need to develop a noninvasive method to predict the PD-1/PD-1 expression status in HCC preoperatively.

In recent years, rapid developments of artificial intelligence have led to its playing an important role in personalized precision medicine. Radiomics is a new technology that can transform potential pathophysiological information in medical images into high-dimensional quantitative imaging features^24,25^. It can help with tumor classification and prediction by finding relationships between quantitative imaging features and clinical and genetic data. Magnetic resonance imaging (MRI)-based radiomics has been applied in many clinical areas^26,27^, but few studies have addressed the value of radiomics models based on multisequence MRI in preoperative predicting PD-1/PD-L1 expression in HCC patients. This paper mainly explores the effectiveness of the preoperative prediction of PD-1/PD-L1 expression status in HCC patients based on multisequence MRI radiomics features.

## Materials and methods

### Patients

The present study was conducted in accordance with the Declaration of Helsinki, and the requirement for informed consent was waived due to the retrospective nature of the study and the anonymous collection of data without any risk for the patient. The study was approved by the Ethics Committee of the Affiliated Hospital of North Sichuan Medical University (No. 2022ER013-1). The preoperative clinical, MRI, and pathological data of patients with postoperative pathologically confirmed HCC who underwent surgical resection at the Affiliated Hospital of North Sichuan Medical University from January 2018 to June 2021 were retrospectively analyzed. The inclusion criteria were as follows: (1) Postoperative pathologically confirmed HCC. (2) Multisequence MRI examination of the upper abdomen performed within 2 weeks before surgery. (3) No prior antitumor therapy. The exclusion criteria were as follows: (1) Incomplete data. (2) Maximum diameter of the lesion less than 2 cm. (3) Combined HCC and intrahepatic cholangiocarcinoma. The included patients were randomly assigned to the training group and validation group in a 7:3 ratio.

### Immunohistochemistry

The expression of PD-1/PD-L1 was detected by immunohistochemistry. Pathological sections were independently evaluated by two doctors, and disagreements were resolved by discussion. Tonsil tissue was used as a positive control. In accordance with published methods, the results of PD-1/PD-L1 immunohistochemical staining were scored^28–30^. PD-1 expression was scored according to the percentage of positive cells and staining intensity. Positive staining was defined as light-yellow to dark-brown staining of the cell membrane or cytoplasm. The entire field of view of each slice was observed under a low-magnification microscope, and then six randomly selected fields of view with lymphocyte aggregation were read under high magnification (400×). The scoring scale for the proportion of positive cells was as follows: < 5%: 0 points; 5–24%: 1 point; 25–49%: 2 points; 50–100%: 3 points. The scoring scale for the staining was as follows: no staining: 0 points; light yellow: 1 point; light brown: 2 points; dark brown: 3 points. The average of the total scores (positive cells + staining) of the six fields of view was calculated. An average score of < 3 was deemed negative for PD-1 expression, and an average score of ≥ 3 was deemed positive for PD-1 expression^17,21^. PD-L1 expression was scored as the proportion of PD-L1 staining in tumor cells. Positive staining was defined as light-yellow to dark-brown staining of the cell membrane or cytoplasm; the proportion of tumor cells stained with PD-L1 was the percentage of stained tumor cells out of all tumor cells in the section. Positive expression was defined as a proportion of positively stained cells ≥ 1%.

### MR image acquisition

Scanning was performed using a Discovery 750 3.0-T superconducting MRI scanner (GE, USA). A 32-channel phased-array surface coil was used for scanning. All study subjects fasted for 4 h before the MRI scan and were taught breathing exercises. Scanning sequence: Axial fat suppression T2-weighted imaging (FS-T2WI), axial dynamic enhanced scanning 3D-LAVA sequence (Table 1). The contrast agent used for dynamic enhancement was Gd-DTPA at a dose of 15–20 mL. A high-pressure syringe was used to inject the contrast agent through a vein on the back of the hand at a rate of 2–2.5 mL/s. The hepatic arterial phase, portal venous phase, and delayed phase were scanned after contrast medium injection.

**Table 1:** Magnetic resonance (MR) imaging scanning sequences and parameters.

Sequence	TR/TE (ms)	FA (°)	Matrix (mm^2^)	FOV (mm^2^)	ST (mm)
BH Ax LAVA-flex	4/2	12	260 × 192	320 × 320–360 × 360	2.6
RTr Ax fs T2	2609/97	110	384 × 384	320 × 320–380 × 380	5
BH Ax LAVA-flex + C	4/2	12	224 × 192	320 × 320–360 × 360	5

TR, repetition time; TE, echo time; FA, flip angle; FOV, field of view; ST, section thickness; LAVA-flex, liver acquisition with volume acceleration-flexible.

### Tumor segmentation and feature extraction

**Figure 1:**
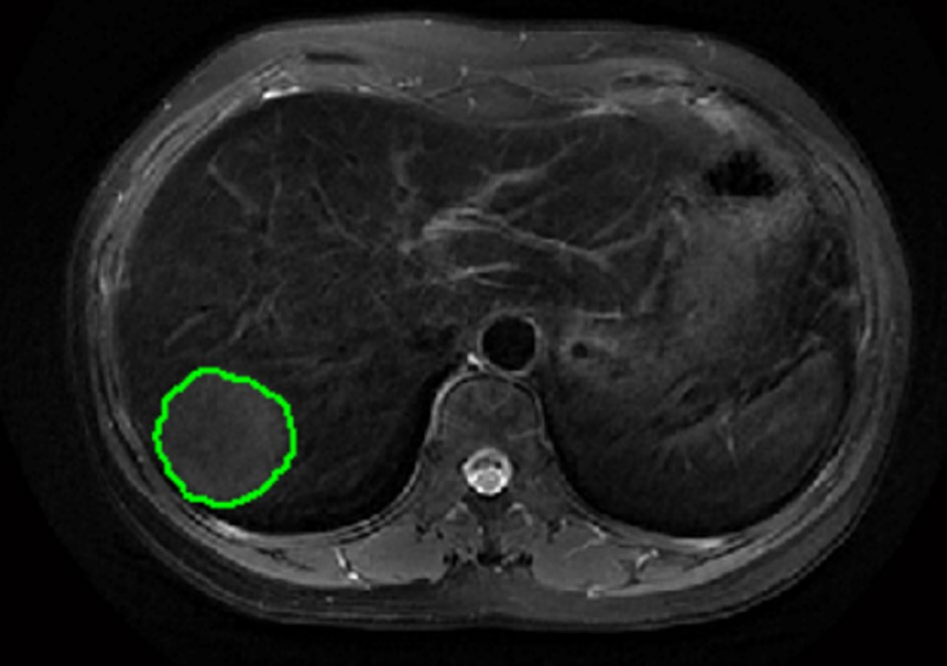
Region of interest delineated along the edge of the HCC lesion.

The volume of the entire tumor was delineated layer by layer along the edge of the lesion as regions of interest on FS-T2W images and axial dynamic-enhanced images in the arterial phase and portal venous phase (Fig. 1). The radiomics features were extracted and divided into four categories: gray-level cooccurrence matrix (GLCM), gray-level run length matrix (GLRLM), intensity histogram, and shape. A dataset of different scan sequences features from FS-T2WI, arterial phase and portal venous phase images was generated. Interobserver agreement was tested on the results recorded by two radiologists (observers 1 and 2) (2 and 5 years of experience, respectively) as a test indicator. The intergroup correlation coefficient (ICC) was used to assess interobserver agreement. When ICC ≥ 0.75, the two observers had good consistency.

### Feature screening and model establishment

In the first step, to eliminate the exponential dimensional differences among the data, all the data were standardized by the z score normalization method. In the second step, features with an ICC < 0.75 were eliminated. The dataset generated by each sequence needed to be checked for consistency. In the third step, the screened stable features were analyzed by a single-factor statistical analytical method (the independent-sample *t* test or Mann‒Whitney* U* test was chosen according to the characteristics of the data distribution). Stable features with statistically significant differences in PD-1/PD-L1 expression were selected (*P* < 0.05). In the fourth step, to avoid overfitting, least absolute shrinkage and selection operator (LASSO) regression analysis was used to select the core radiomic features predicting PD-1/PD-L1 expression. Using the minimum criterion (1 minus standard error), the regularization parameter (λ) of the selected features was adjusted by tenfold cross-validation.

The optimal radiomics features selected from each sequence were used to construct a single-sequence and multisequence radiomics prediction models by logistic regression^31,32^. The radiomic-clinical model was constructed by combining multisequence radiomics features and clinical characteristics. The training set data were used to train the model, and the validation set data were used to validate the model. The predictive performance of the models was evaluated by calculating the area under the receiver operating characteristic (ROC) curve (AUC), sensitivity, specificity, positive predictive value (PPV), negative predictive value (NPV), accuracy, and F1-score of the confusion matrix.

### Statistical analysis

R software (version 4.0.2. https://www.r-project.org/) was used for the statistical analysis in this study. The R packages used included "psych", "glmnet", and "pROC". "psych" was used to assess the intergroup agreement for the radiomics characteristics; "glmnet" was used to perform LASSO regression analysis; "pROC" was used to draw the ROC curves. Quantitative data are described as the median. The Shapiro‒Wilk test was used to judge distribution normality for these variables, and the Bartlett test was used to judge homogeneity of variance. When both tests were satisfied, the independent-sample t test was used; otherwise, the Mann‒Whitney U test was used for comparisons between groups. Categorical variables are described as percentages, and the chi-squared test was used for comparisons between groups. A two-tailed *P* value < 0.05 was considered statistically significant.

## Results

**Figure 2:**
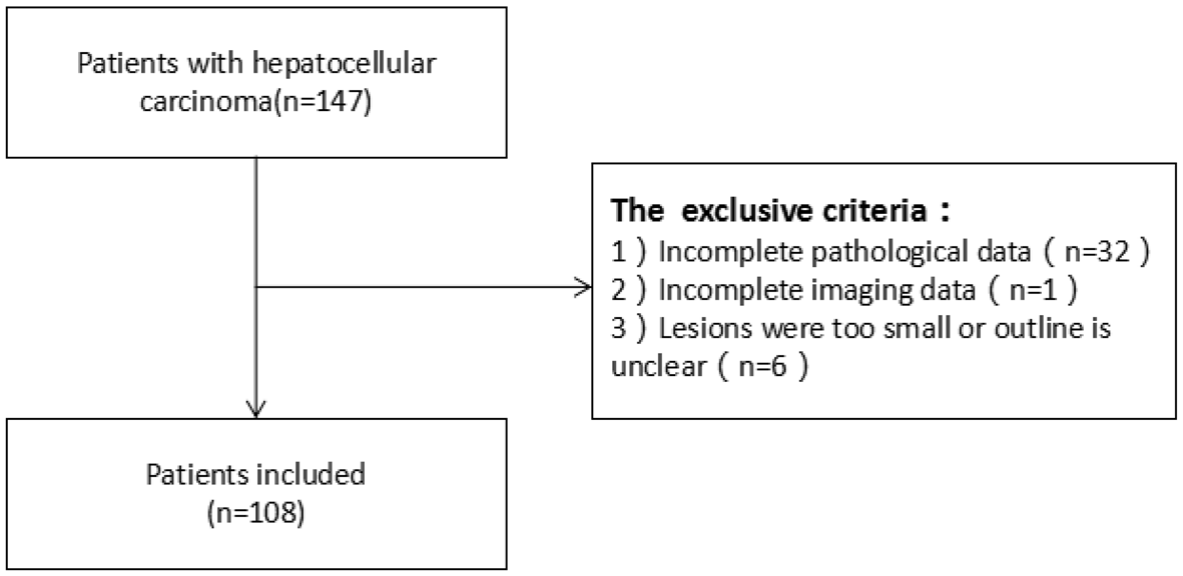
Flow chart of patient inclusion for the present study.

**Figure 3:**
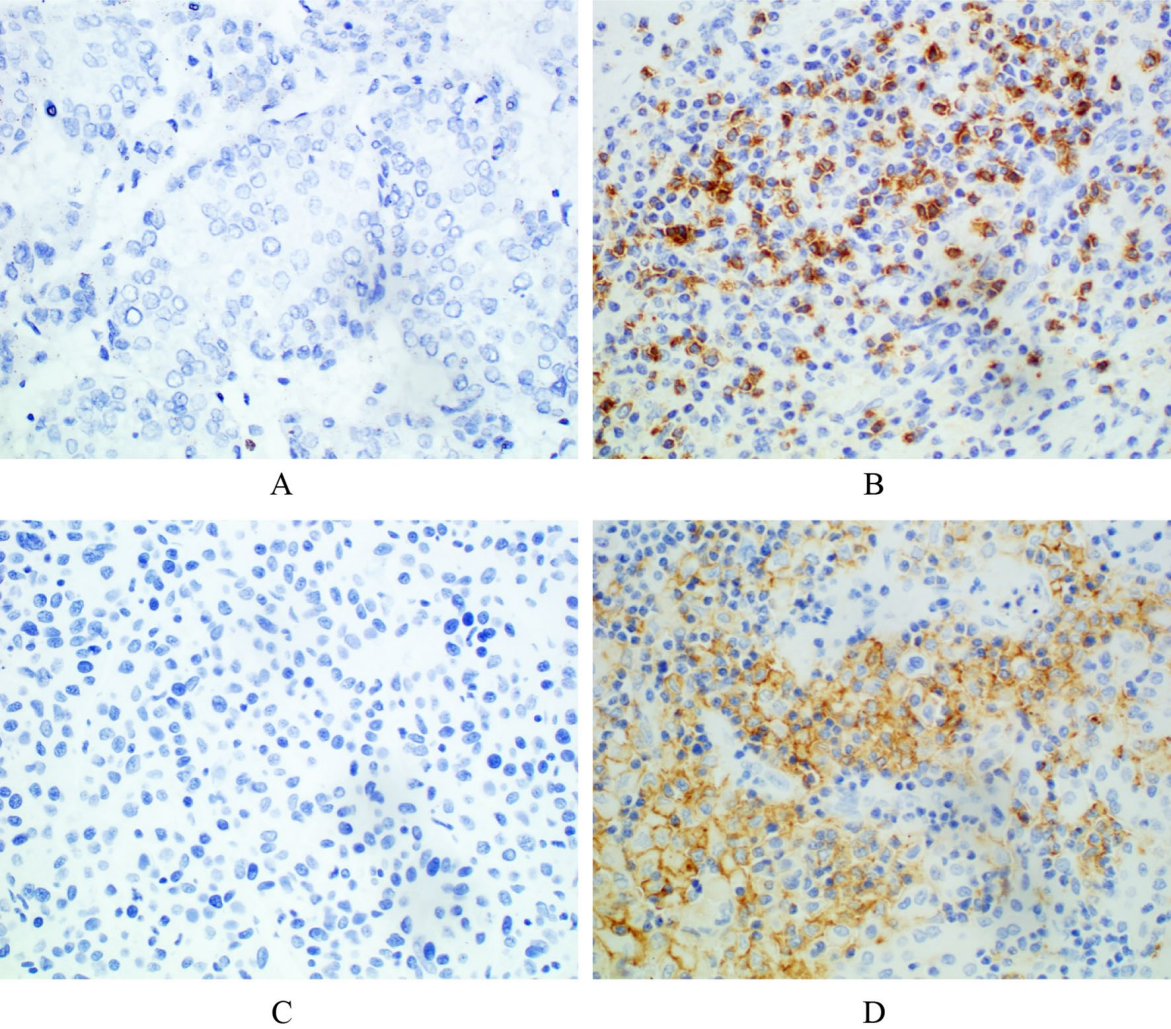
Immunohistochemical staining of HCC tissues for PD-1 and PD-L1 expression (×400). (**A**) Negative PD-1 expression. (**B**) Positive PD-1 expression. (**C**) Negative PD-L1 expression. (**D**) Positive PD-L1 expression.

In all, 147 patients were considered for enrollment, and of these, 108 patients met the criteria and were included in this study (Fig. 2). Among the 108 enrolled patients, 95 were male and 13 were female; 81 patients had liver cirrhosis, and 37 patients were diagnosed with multiple tumors. The maximum tumor diameter ranged from 2.0 to 20.1 cm. Positive PD-1 expression was observed in 43 patients, while negative PD-1 expression was observed in 65 cases.A total of 34 patients had PD-L1-positive expression, and 74 had PD-L1-negative expression. Among the clinical characteristics, the presence of satellite nodules served as an independent predictor of PD-L1 expression (Fig. 3; Tables 2, 3).

**Table 2:** Preoperative clinical characteristics of PD-1(+) and PD-1(−) patients in the training and validation groups.

Clinical characteristics	Training		*P* value	Validation		*P* value
	PD-1(−) (*n* = 45)	PD-1(+) (*n* = 30)		PD-1(−) (*n* = 20)	PD-1(+) (*n* = 13)	
Age	55 (29–73)	53.5 (30–72)	0.549	51.5 (23–68)	53 (37–70)	0.372
Sex (%)			0.067			0.289
Male	29 (96.7)	36 (80)		17 (85)	13 (100)	
Female	1 (3.3)	9 (20)		3 (15)	0 (0)	
AFP (ng/mL)						
< 20	18 (40)	10 (33.3)		8 (40.0)	5 (38.5)	
20–400	11 (24.4)	8 (26.7)	0.585	2 (10.0)	4 (30.8)	0.587
> 400	16 (35.6)	12 (40.0)	0.959	10 (50)	4 (30.8)	0.125
AST (U/L)			0.301			0.349
≤ 44	25 (55.6)	13 (43.3)		6 (30.0)	6 (46.2)	
> 44	20 (44.4)	17 (56.7)		14 (70.0)	7 (53.8)	
ALT (U/L)			0.918			0.437
≤ 50	32 (71.1)	21 (70.0)		12 (60.0)	6 (46.2)	
> 50	13 (28.9)	9 (30.0)		8 (40.0)	7 (53.8)	
ALB (g/L)			0.172			0.078
≥ 40	26 (57.8)	22 (73.3)		9 (45.0)	10 (76.9)	
< 40	19 (42.2)	8 (26.7)		11 (55.0)	3 (23.1)	
TBIL (μmol/L)			0.840			0.948
≤ 20	31 (68.9)	20 (66.7)		11 (55.0)	7 (53.8)	
> 20	14 (31.1)	10 (33.3)		9 (45.0)	6 (46.2)	
PT (s)			0.653			0.513
≤ 14.5	34 (75.6)	24 (80.0)		15 (75.0)	11 (84.6)	
> 14.5	11 (24.4)	6 (20.0)		5 (25.0)	2 (15.4)	
Diameter (cm)			0.850			0.532
0–5	20 (44.4)	14 (46.7)		7 (35)	6 (46.2)	
≥ 5	25 (55.6)	16 (53.3)		13 (65.0)	7 (53.8)	
Hepatitis B			0.871			0.537
Absent	4 (8.9)	3 (10.0)		3 (15.0)	1 (7.7)	
Present	41 (91.1)	27 (90)		17 (85.0)	12 (92.3)	
Cirrhosis			0.116			0.833
Absent	15 (33.3)	5 (16.7)		4 (20.0)	3 (23.1)	
Present	30 (66.7)	25 (83.3)		16 (80.0)	10 (76.9)	
Ascites			0.772			0.948
Absent	27 (60)	19 (63.3)		11 (55.0)	7 (53.8)	
Present	18 (40)	11 (36.7)		9 (45.0)	6 (46.2)	
Intravascular tumor thrombus			0.243			0.591
Absent	38 (84.4)	22 (73.3)		12 (60.0)	9 (69.2)	
Present	7 (15.6)	8 (26.7)		8 (40.0)	4 (30.8)	
Satellite nodule			0.282			0.216
Absent	26 (57.8)	21 (70.0)		13 (65.0)	11 (84.6)	
Present	19 (42.2)	9 (30.0)		7 (35.0)	2 (15.41)	

AFP, α-fetoprotein; AST, aspartate aminotransferase; ALT, alanine aminotransferase; ALB, albumin; TBIL, total bilirubin; PT, prothrombin time; PD-1, programmed cell death protein 1.

**Table 3:** Preoperative clinical characteristics of PD-L1(+) and PD-L1(−) patients in the training and validation groups.

Clinical characteristics	Training		*P* value	Validation		*P* value
	PD-L1(−) (*n* = 51)	PD-L1(+) (*n* = 23)		PD-L1(−) (*n* = 23)	PD-L1(+) (*n* = 11)	
Age	52 (23–72)	53 (32–72)		54 (33–73)	55 (34–69)	
Sex (%)			0.768			0.999
Male	43 (84.3)	20 (87.0)		21 (91.3)	11 (100)	
Female	8 (15.7)	3 (13.0)		2 (8.7)	0 (0)	
AFP (ng/mL)						
< 20	19 (37.3)	13 (56.5)		7 (30.4)	2 (18.2)	
20–400	11 (21.6)	4 (17.4)	0.136	6 (26.1)	4 (36.4)	0.564
> 400	21 (41.2)	6 (26.1)	0.746	10 (43.5)	5 (45.5)	0.734
AST (U/L)			0.573			0.914
≤ 44	23 (45.1)	12 (52.2)		10 (43.5)	5 (45.5)	
> 44	28 (54.9)	11 (47.8)		13 (56.5)	6 (54.5)	
ALT (U/L)			0.277			0.730
≤ 50	31 (60.8)	17 (73.9)		16 (69.6)	7 (63.6)	
> 50	20 (39.2)	6 (26.1)		7 (30.4)	4 (36.4)	
ALB (g/L)			0.747			0.164
≥ 40	29 (56.9)	14 (60.9)		18 (78.3)	6 (54.5)	
< 40	22 (43.1)	9 (39.1)		5 (21.7)	5 (45.5)	
TBIL (μmol/L)			0.994			0.540
≤ 20	31 (60.8)	14 (60.9)		17 (73.9)	7 (63.6)	
> 20	20 (39.2)	9 (39.1)		6 (26.1)	4 (36.4)	
PT (s)			0.413			0.955
≤ 14.5	40 (78.4)	16 (69.6)		19 (82.6)	9 (81.8)	
> 14.5	11 (21.6)	7 (30.4)		4 (17.4)	2 (18.2)	
Diameter (cm)			0.042*			0.394
0–5	20 (39.2)	15 (65.2)		7 (30.4)	5 (45.5)	
≥ 5	31 (60.8)	8 (34.8)		16 (69.6)	6 (54.5)	
Hepatitis B			0.308			0.693
Absent	3 (5.9)	3 (13.0)		3 (13.0)	2 (18.2)	
Present	48 (94.1)	20 (87.0)		20 (87.0)	9 (81.8)	
Cirrhosis			0.822			0.550
Absent	10 (19.6)	4 (17.4)		8 (34.8)	5 (45.5)	
Present	41 (80.4)	19 (82.6)		15 (65.2)	6 (54.5)	
Ascites			0.303			0.400
Absent	29 (56.9)	16 (69.6)		14 (60.9)	5 (45.5)	
Present	22 (43.1)	7 (30.4)		9 (39.1)	6 (54.5)	
Intravascular tumor thrombus			0.397			0.955
Absent	35 (68.6)	18 (78.3)		19 (82.6)	9 (81.8)	
Present	16 (31.4)	5 (21.7)		4 (17.4)	2 (18.2)	
Satellite nodule			0.012*			0.071
Absent	28 (54.9)	20 (87.0)		13 (56.5)	10 (90.9)	
Present	23 (45.1)	3 (13.0)		10 (43.5)	1 (9.1)	

AFP, α-fetoprotein; AST, aspartate aminotransferase; ALT, alanine aminotransferase; ALB, albumin; TBIL, total bilirubin; PT, prothrombin time; PD-L1, programmed cell death protein ligand 1.

*Statistically significant at *P* < 0.05.

**Figure 4:**
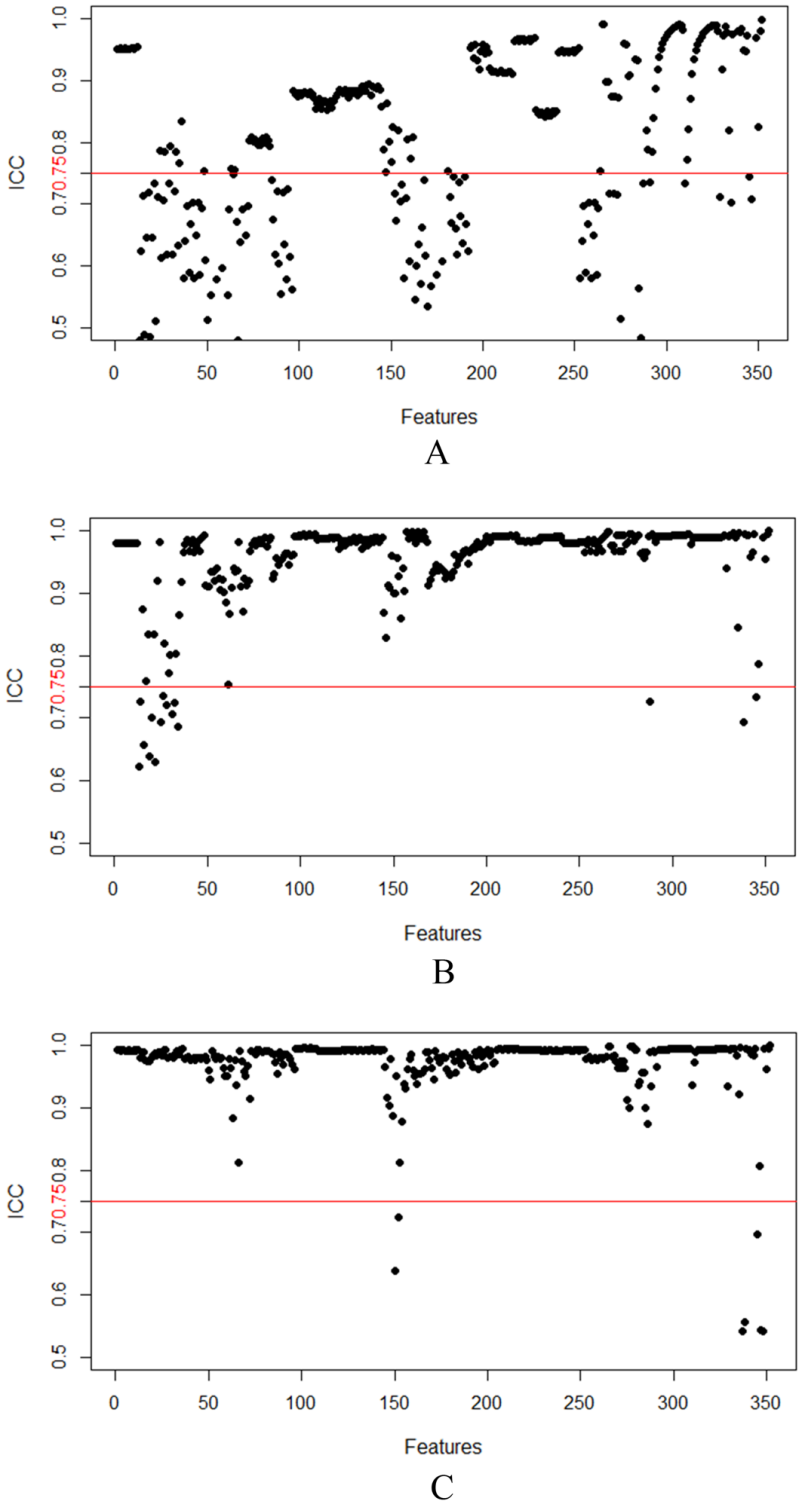
Stability assessment of extracted MRI radiomics features by ICC. (**A**) FS-T2WI; (**B**) Arterial phase; (**C**) Portal venous phase.

**Figure 5:**
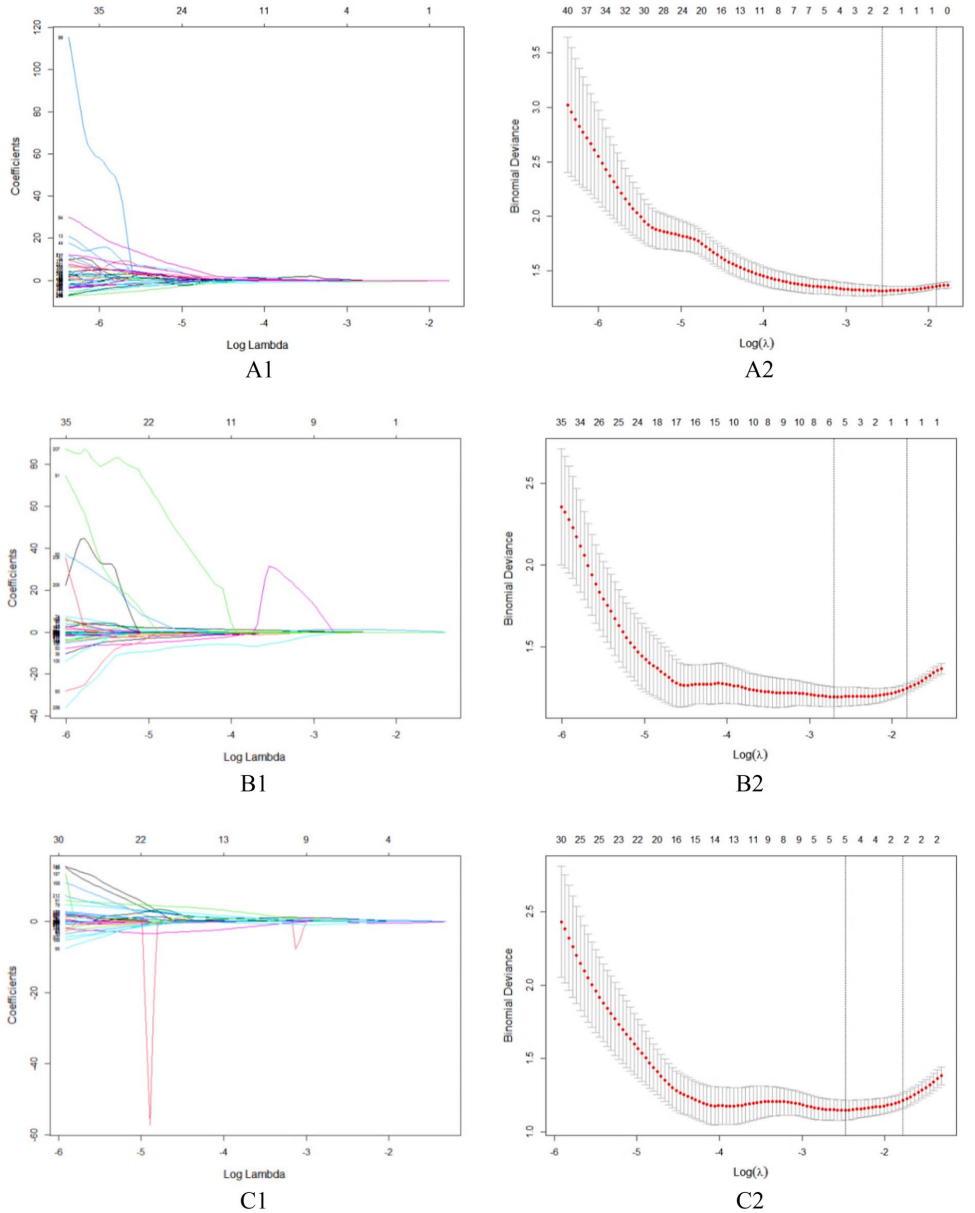
Feature selection using LASSO regression to predict PD-1 expression. (**A1**–**A2**) FS-T2WI; (**B1**–**B2**) Arterial phase; (**C1**–**C2**) Portal venous phase.

**Figure 6:**
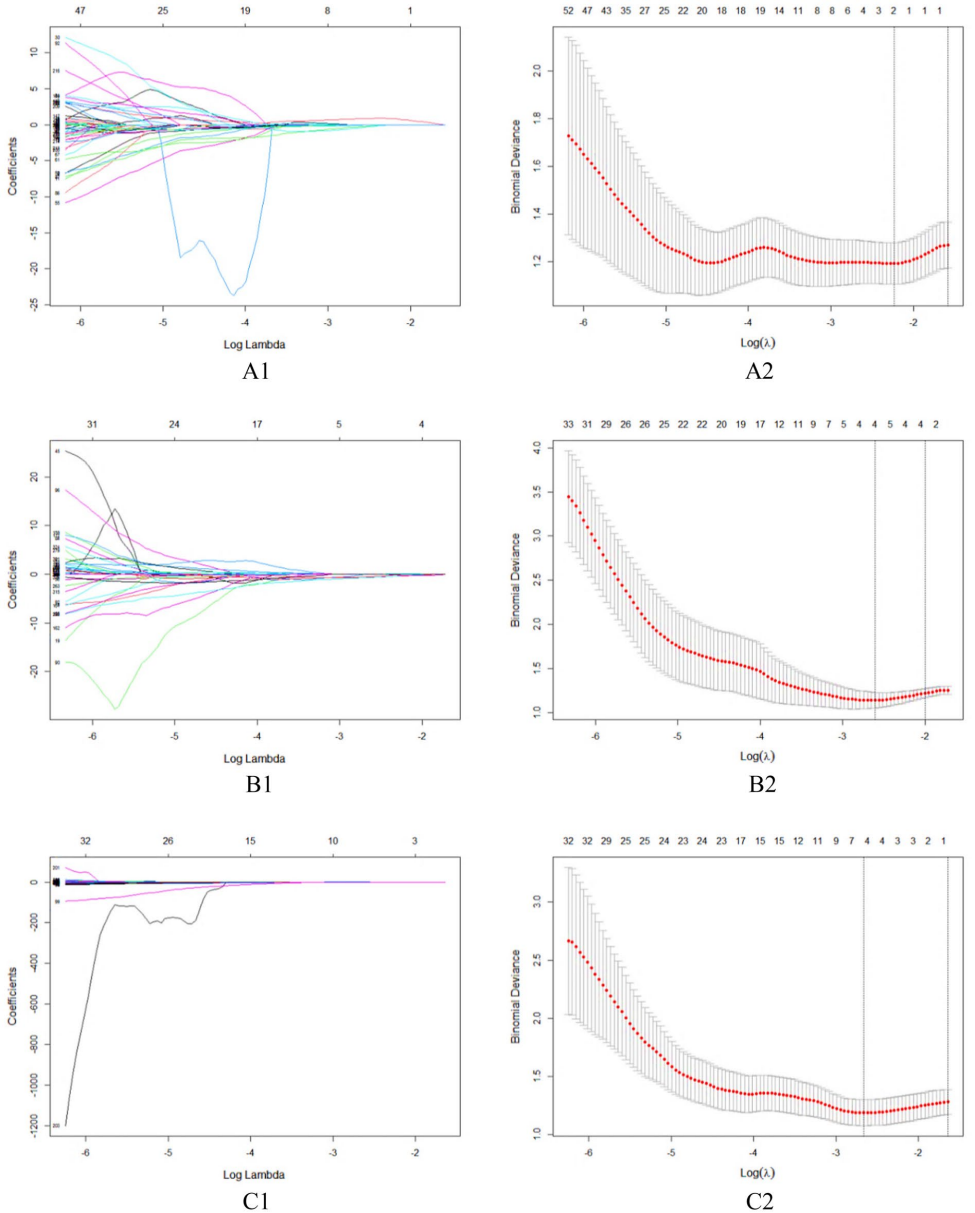
Feature selection using LASSO regression to predict PD-L1 expression. (**A1**–**A2**) FS-T2WI; (**B1**–**B2**) Arterial phase; (**C1**–**C2**) Portal venous phase.

We extracted 352 features from the FS-T2WI, arterial phase, and portal venous phase datasets, and features with an ICC score lower than 0.75 were excluded. The remaining features were further analyzed (Fig. 4). In the analysis of PD-1 expression status, there were 221, 333, and 331 features in the FS-T2WI, arterial phase, and portal venous phase datasets, respectively, which were significantly different according to the independent-sample *t* test or Mann‒Whitney* U* test (*P* < 0.05). LASSO regression selected two, six, and five optimal features from the statistically significant radiomics features, respectively (Fig. 5). In the analysis of PD-L1 expression status, according to the independent-sample *t* test or Mann‒Whitney* U* test, there were 221, 326, and 344 features in the FS-T2WI, arterial phase, and portal venous phase datasets, respectively, that were significantly different between the PD-L1-negative and PD-L1-positive status groups (*P* < 0.05). LASSO regression selected two, four, and six optimal features from the statistically significant radiomics features, respectively (Fig. 6).

**Figure. 7:**
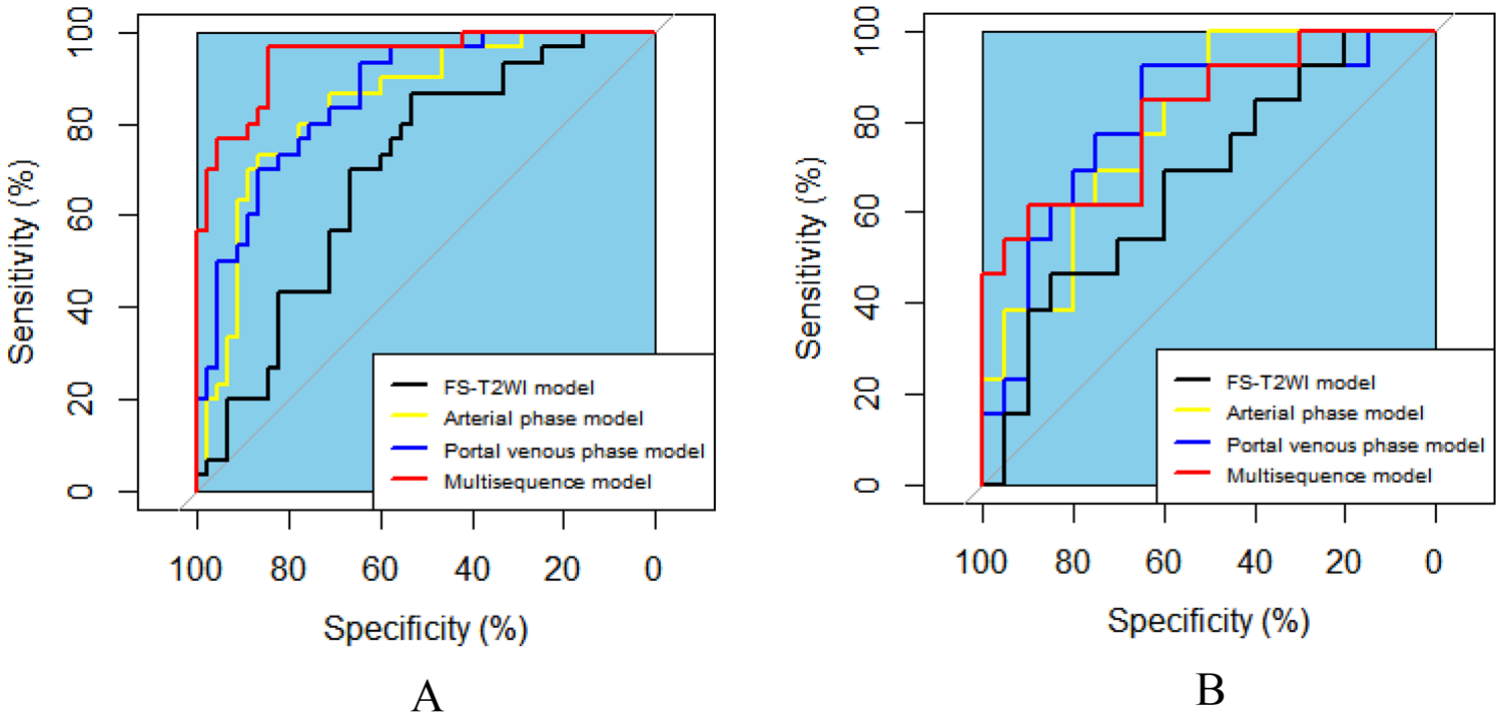
ROC curves showing the performance of the FS-T2WI, arterial phase, portal venous phase, and multisequence models in predicting PD-1 expression. (**A**) Training set. (**B**) Validation set.

In the analysis of PD-1 expression status, as shown in the above steps, two, six, and five features screened from the FS-T2WI, arterial phase and portal venous phase datasets, respectively, were used to construct the FS-T2WI, arterial phase and portal venous phase radiomics models. These features were also synthesized to construct the multisequence model. The predictive performance of the models was evaluated by the AUC, sensitivity, specificity, PPV, NPV, accuracy, and F1-score. The AUC values of the four radiomics models FS-T2WI, arterial phase, portal venous phase, and multisequence were 0.696, 0.843, 0.863, and 0.946, respectively, in the training group and 0.669, 0.792, 0.800, and 0.815 in the validation group. The predictive performance of the multisequence model was better than that of the FS-T2WI, arterial phase, and portal venous phase models (Table 4, Fig. 7).

**Table 4:** Predictive performance of each model for HCC PD-1 expression.

Cohort	Prediction model	AUC	Sen	Spe	PPV	NPV	ACC	F1-score
Training cohort	FS-T2WI model	0.696	0.533	0.711	0.552	0.696	0.640	0.542
	Arterial phase model	0.843	0.800	0.756	0.686	0.850	0.773	0.738
	Portal venous phase model	0.863	0.767	0.778	0.670	0.833	0.773	0.730
	Multisequence model	0.946	0.800	0.889	0.828	0.867	0.870	0.814
Validation cohort	FS-T2WI model	0.669	0.692	0.550	0.500	0.733	0.606	0.581
	Arterial phase model	0.792	0.461	0.800	0.600	0.696	0.667	0.522
	Portal venous phase model	0.800	0.693	0.800	0.692	0.800	0.756	0.692
	Multisequence model	0.815	0.615	0.650	0.533	0.722	0.636	0.571

AUC, area under the receiver operating characteristic curve; ACC, accuracy; Sen, sensitivity; Spe, specificity; PPV, positive predictive value; and NPV, negative predictive value.

**Figure 8:**
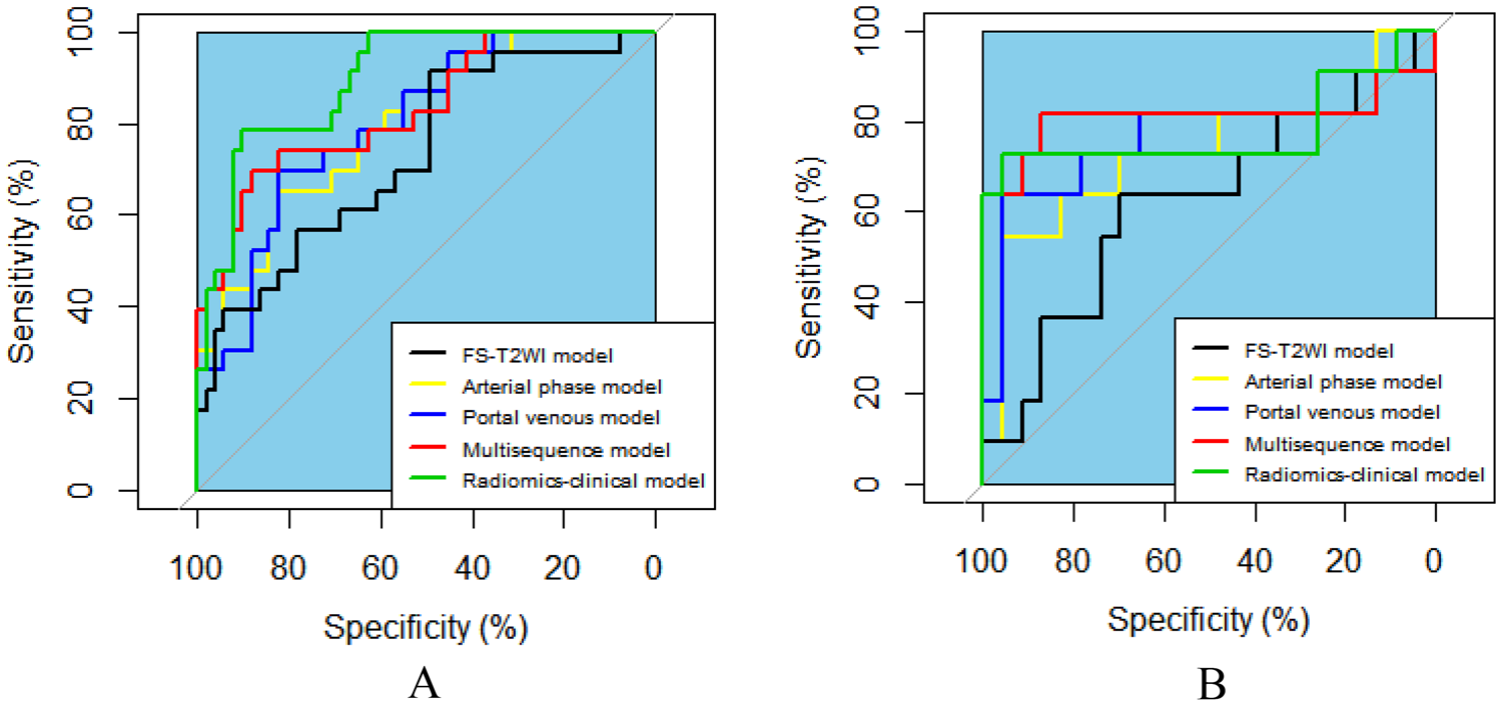
ROC curves showing the performance of the FS-T2WI, arterial phase, portal venous phase, multisequence and radiomics-clinical models in predicting PD-L1 expression. (**A**) Training set. (**B**) Validation set.

In the analysis of PD-L1 expression status, the two, four, and six features screened from the FS-T2WI, arterial phase, and portal venous phase datasets, respectively, in the above steps were used to construct the FS-T2WI, arterial phase, and portal venous phase radiomics models. These features were then synthesized to construct the multisequence model, and the predictive performance of all models was evaluated using the above metrics. The AUC values of the FS-T2WI, arterial phase, portal venous phase, multisequence and radiomic-clinical models were 0.731, 0.800, 0.800, 0.831 and 0.898, respectively, in the training group and 0.621, 0.743, 0.771, 0.810 and 0.779 in the validation group. The combined models had better predictive performances (Table 5, Fig. 8).

**Table 5:** Predictive performance of each model for HCC PD-L1 expression.

Cohort	Prediction model	AUC	Sen	Spe	PPV	NPV	ACC	F1-score
Training cohort	FS-T2WI model	0.731	0.391	0.902	0.642	0.767	0.743	0.486
	Arterial phase model	0.800	0.435	0.922	0.714	0.783	0.770	0.541
	Portal venous phase model	0.800	0.435	0.882	0.625	0.776	0.743	0.513
	Multisequence model	0.831	0.565	0.902	0.722	0.821	0.797	0.634
	Radiomics-clinical model	0.898	0.652	0.922	0.790	0.855	0.838	0.714
Validation cohort	FS-T2WI model	0.621	0.272	0.870	0.500	0.714	0.676	0.353
	Arterial phase model	0.743	0.545	0.956	0.857	0.815	0.824	0.667
	Portal venous phase model	0.771	0.364	0.957	0.800	0.759	0.765	0.500
	Multisequence model	0.810	0.454	1.000	1.000	0.793	0.824	0.625
	Radiomics-clinical model	0.779	0.636	1.000	1.000	0.852	0.882	0.778

AUC, area under the receiver operating characteristic curve; ACC, accuracy; Sen, sensitivity; Spe, specificity; PPV, positive predictive value; and NPV, negative predictive value.

## Discussion

The PD-1/PD-L1 pathway plays a key role in the development of chronic liver infection, tumor immune response evasion, and tumor microenvironment formation^33^. Previous studies have indicated that the overexpression of PD-1/PD-L1 in HCC patients is closely related to their poor prognosis and tumor recurrence^14,17,34–40^; PD-1/PD-L1 expression may serve as a biomarker for predicting ICI treatment response in HCC patients^13,33,36,41,42^.

Radiomics extracts high-dimensional data from traditional medical images that cannot be assessed by the naked eye and has a strong correlation with heterogeneity at the cellular level^43^. Recently, radiomics has been applied to the analysis of PD-1/PD-L1 expression in lung cancer, breast cancer, etc.^30,44–50^, but has rarely been applied to studies of the PD-1/PD-L1 expression status in HCC^51–53^. Tian et al.^52^ extracted radiomics and deep learning features based on preoperative T2WI sequences and used an integrated model to predict the expression of PD-L1 in HCC tissues. The results showed that the AUC of the radiomics-based model was 0.794 ± 0.035; the model combining radiomics and deep learning features achieved the best predictive performance, with an AUC value of 0.897 ± 0.084. However, they only studied a single sequence, T2WI, and did not incorporate other sequences, such as contrast-enhanced MRI. In the present study, we established a multisequence MRI-based radiomics model by integrating the radiomic features from FS-T2WI, contrast-enhanced arterial-phase, and portal venous-phase sequence to predict the expression of PD-1 and PD-L1, with AUCs of 0.946 and 0.831 in the training group, respectively, demonstrating an improvement in the prediction performance. Previous HCC radiomics studies have shown the advantages of multisequence combined models^54–57^. Because different sequences reveal different information about the tumor, the multisequence combined radiomics model had the best predictive performance. The conclusion of the present study is consistent with the literature^54–57^.

In this study, 13 and 12 core radiomic features were extracted from FS-T2WI, arterial-phase, and portal venous-phase MRI images to construct radiomics models for predicting PD-1 and PD-L1 expression, respectively. The selected radiomics features were mainly derived from GLCM features, a group of texture features that can be used to evaluate tumor heterogeneity by reflecting the relationship between adjacent voxels^58,59^. This result is similar to that of some previous studies^52,53,60,61^. Zhang et al.^60^ converted MRI radiomics features of liver tumors into a quantitative Radscore for the preoperative prediction of PD-1/PD-L1 expression, and they found that the radiomics features associated with PD-1/PD-L1 expression were mainly GLCM features. Second, morphological features were also closely related to the expression of PD-L1. Max3Ddiameter and SurfaceAreaDensity are the most relevant of these features: Max3Ddiameter is the longest 3D diameter of the tumor mass, and SurfaceAreaDensity is its surface area density. The morphological features reflect the external manifestation of the tumor profile. Wen et al.^47^ believed that positive PD-L1 expression was closely related to tumor shape (*P* = 0.006). Histogram analysis describes the global distribution of gray levels in an image and can be used to assess tumor heterogeneity^62^. Shi et al.^63^ found that the histogram index extracted from the IVIM parameter map could predict the expression of Ki-67.

In the field of oncology, research results have been inconsistent regarding the relationship between PD-1/PD-L1 and clinical risk factors, and there is great debate about this relationship^37,64–67^. In the study of Hu et al.^65^, high PD-L1 expression in HCC was associated with tumor size (*P* = 0.033) and the presence of satellite nodules (*P* = 0.018). Li et al.^37^ showed that PD-L1 overexpression was significantly associated with tumor differentiation, history of hepatitis, elevated alpha-fetoprotein (AFP), and tumor-infiltrating lymphocytes and was not significantly associated with the maximum tumor diameter (*P* = 0.07) or tumor number (*P* = 0.54). A meta-analysis by Zhang et al.^67^ showed that PD-1 expression was significantly correlated with age (*P* = 0.023) and AFP (*P* = 0.000). Among all the clinical factors analyzed in this study, only the maximum tumor diameter and tumor number were associated with PD-L1 expression, which is consistent with the findings of Hu et al.^65^. Multivariate analysis revealed that the presence of satellite nodules was an independent predictor of PD-L1 expression. No other clinical factors were found to be associated with PD-L1 expression, nor was PD-1 expression found to be associated with any relevant clinical indicator.

This study has the following limitations. First, the sample was small. Because many HCC patients who did not undergo surgical resection or MRI scans were excluded, there may be potential selection bias. Second, the study used data from a single center. The results must be externally validated in other centers. Third, other MRI sequences, such as diffusion-weighted imaging, were not analyzed in this study, and therefore, information from other sequences was ignored. Finally, this study did not develop a prediction model including genetic variables. A combined model for predicting PD-1/PD-L1 expression should be constructed by combining multisequence radiomics features and clinical and genetic characteristics in the future.

In summary, the radiomics model based on multisequence MRI has potential in predicting the preoperative expression of PD-1 and PD-L1 in HCC, which could become an imaging biomarker for ICI treatment.

## Supplementary Information

Supplementary Information.

## Data Availability

All data generated or analyzed during this study are included in this published article and its supplementary information files.
